# Secrecy, self-blame and risks for social exclusion—Family members’ experiences of gambling problems in Japan

**DOI:** 10.3389/fpsyt.2022.940397

**Published:** 2022-10-13

**Authors:** Naoko Takiguchi, Yuko Kawanishi, Eva Samuelsson

**Affiliations:** ^1^Department of Sociology, Otani University, Kyoto, Japan; ^2^College of Global Communication, J.F. Oberlin University, Tokyo, Japan; ^3^Department of Social Work, Stockholm University, Stockholm, Sweden; ^4^Department of Public Health Sciences, Stockholm University, Stockholm, Sweden

**Keywords:** shame, Japanese gambling policies, self-blame, addiction, social exclusion, family, interviews

## Abstract

The predominant gambling policy to respond to the adverse consequences of excessive gambling has been the Reno Model, which places the responsibility for gambling-caused problems on gamblers themselves. The newly implemented Japanese gambling policy, which shares basic premises with the Reno Model, focuses on the individual pathology of gamblers. However, this model lacks other critical perspectives: environmental and structural factors. To fully understand the harms caused by gambling; it is important to also pay attention to the negative consequences for affected others. In this brief report, we explore family members’ experiences of gambling problems within the specific context of the Japanese gambling policy. Interviews with family members reveal self-stigma of being bad parents which elicits shame and efforts to maintain secrecy, as well as public stigma involving labeling, isolation, risks of status loss, social exclusion and discrimination. The focus on individual pathology in Japanese legislation as well as in public and professional perception, reinforces self-blame, anxiety, and remorse on the part of affected family members. When contrasted with the lived experiences of gamblers’ family members, the inconsistencies and unreasonableness of the individual pathology paradigm in Japanese gambling policy become evident. It is necessary to shift the focus of gambling policies from individual to socio-political-cultural factors, investigating how these factors influence gambling-caused harm, especially in the Japanese context.

## Introduction

Excessive gambling affects various domains of the lives of gamblers, their family members and the general public, producing harm such as multiple debts, job loss, marital conflict, domestic violence, child abuse, poor health, homelessness, attempted suicide, and crime (1–3). Negatively affected people include spouses, parents and children who never had any personal gambling experiences (4–6) but are devastated by the gambling of their loved ones. The harms from gambling for family members and communities has, however, been poorly recognized (7) and understudied (8, 9) especially in Japan. To fully understand the harms caused by gambling; it is important to also pay attention to the negative consequences for affected others. In this brief report, we explore family members’ experiences of gambling problems within the specific context of the Japanese gambling policy.

Gambling is legally banned in Japan, and could according to the penal code be punished with fines or imprisonment (10). However, despite the punitive risk, gambling opportunities are pervasive. The latest national prevalence survey revealed that 45% of male and 23% of female respondents had gambled during the past year, including lotteries. Past year gambling disorders affected 2.2% of Japanese adults: 3.7% of males and 0.7% of females (11). Despite the penal code, many Japanese thus engage in gambling, and certain forms of state-approved gambling are permitted for what is called the “public good” in order to collect revenues from government-controlled gambling. Examples include state-regulated betting: national and provincial horse racing; boat, bicycle and motorcycle racing; lotteries; and wagering on professional soccer. Unlike Chinese culture, where family members and friends get together and enjoy table games, e.g., to celebrate the Chinese New Year (12), gambling is in Japanese culture perceived as something risky (13). The most problematic gambling form and the largest of the total amounts of wagering money derives from privately operated electronic gambling machines (EGMs) called *pachinko* and *pachislot*.^1^

*Pachinko* and *pachislot* parlors are ubiquitous across Japan: near train stations, along bustling streets, in urban suburbs and rural towns. Conspicuous glittering signs enticed over seven million gamblers in 2020, even though most parlors were closed for more than 1 month due to COVID-19 (14). Almost 60% of the world’s EGMs absorbed US $108 billion of the wagering money in 2020 (14, 15). People may wonder why such a vast private gambling market exists in Japan while the penal code prohibits gambling. According to legislation, *pachinko* and *pachislot* parlors can operate in Japan because they are classified as “amusement” (16). *Pachinko* and *pachislot* gamblers receive a “prize” (with little monetary value) for their winnings, which they can exchange for cash at a location outside the parlor. The parlor has nothing to do with this exchange. Using this loophole, the *pachinko* and *pachislot* is not regulated as gambling (17). Recent research shows that accessibility to parlors is associated with gambling problems among men and people in low-income areas (18), but restriction of accessibility to reduce gambling harm among vulnerable groups has not been up for discussion in Japan.

Until recently, almost no policies existed that addressed gambling-caused problems in Japan. In 2018, a new law was passed that would allow specially designated areas to open casinos by late 2020s, although, to be clear, this does not mean the legalization of gambling throughout Japan. Integrated resorts including casinos are planned to open in at least two prefectures in Japan (Osaka and Nagasaki) in the upcoming years, despite the strong opposition from the public [64% against, 77% among women (19)] with fear of “more gambling addicts” and “worsened security” as the main reasons (20). In 2019, the government introduced policies such as The Basic Plan to Promote Measures to Address Addiction of Gambling, and so forth^2^ (21).

The most general policy to address gambling problems is based on the Reno Model, which places the responsibility for gambling-caused harm primarily on gamblers themselves (22). Recently, criticism has increased regarding the Reno Model, which neglects the influence of the gambling environment and the risky nature of gambling products (23, 24). The concept of “responsible gambling” generates shame and a sense of guilt for both gamblers who fail to control their gambling behavior and their family members who fail to stop their loved ones from gambling (17, 25–28).

This particular Japanese gambling policy does not explicitly promote the concept of responsible gambling; however, the policy can be identified as a type of the Reno Model (22). The policy focuses on people with gambling addiction, the social impact of their gambling addiction and gamblers’ family members. The gambling industry is highly encouraged to give financial support to treatment facilities. As for the gamblers’ protection, gamblers, and their family members may submit self-exclusion applications. A limit-setting system will be introduced for all forms of online racing betting 2022 (21). The policy expects all the stakeholders, including gambling industries, to collaborate. Therefore gambling industry representatives are included in the stakeholders’ meeting organized by the Japanese Secretariat where “measures on the addiction of gambling and so forth” are discussed (29).

Gambling addiction is by the Ministry of Health Labor and Welfare conceptualized as a treatable disease from which anyone might suffer and recover. People are advised to become aware of the early signs of addiction (30). Little is expected of the gambling industry to reduce gambling-related harm, provide practical gambler-protection tools or restrict the number of EGMs in the area. The gambling industry recommends that people gamble in a “moderate” or “appropriate” way, known as *tekidoni.* However, no helpful information is offered to inform gamblers on whether or not they are playing in a *tekidoni*-like manner (31). The responsibility falls on the gambler and family members to recognize early gambling addiction signs and submit self-exclusion applications. The gambling policy relies on gamblers’ personal responsibility and originates from basic Japanese health policies.

For instance, in 2002, the Japanese Health Promotion Act (32) decreed the responsibility of citizens for their own health by stating that “people must endeavor to deepen their interest in and understanding of the importance of healthy lifestyles, and to both be aware of the state of their own health and improve their health throughout the course of their lives.”^3^ Shibata (33), a Japanese expert on social welfare, argues that since the 1950s, the Japanese government has repeatedly reinforced personal responsibility in health-related policies. People are obliged to prevent the increase of medical fees by paying “attention to their own health and take care of themselves so as not to suffer from any disease” through, i.e., physical exercise, healthy meals, and non-smoking (33), p. 39.

If people are responsible for their health, how does the Japanese public perceive people with gambling addiction? A recent survey shows that the public consensus is that gamblers are responsible for the consequences of their gambling behavior (11). To the question: “Are the individuals responsible for their gambling-related suffering?,” 72.6% replied “yes,” while the equivalent regarding depression (8.8%) and diabetes (28.5%) was considerably lower.

The national Japanese prevalence rate for gambling, especially the 3.7% for males (11), is comparatively higher than in other countries (34), yet seeking help for gambling problems is rare. To the multiple-choice question, “Have you ever sought help for your gambling problems,” 94.7% of those who had lifetime gambling experiences replied they had never sought help. Among those who sought help, 4.6% had sought help from family and friends, 0.1% from public health centers and 0.1% from clinics and hospitals (11).

Even professionals engaged in support services equate gambling-caused problems with gamblers, and they believe gamblers should be aware of their problems and make an individual effort to stop gambling. To the question, “What is needed to support those who have gambling problems?,” most professionals suggested using the framework of individual pathology and treatment. That is to say, the person’s behavior negatively affects family members and society. The person is sick and must undergo treatment, but most do not. For example, quotes from professionals working in the support service (11) reveals that gamblers “do not acknowledge their problems, that they “need to become aware that gambling addiction is a disease,” and that they should “admit themselves to a rehabilitation center.” Family members are in turn expected “to gain correct knowledge on gambling addiction.” Policy or prevention measures including social or environmental factors are not mentioned in the professionals’ comments.

The focus on individual pathology is also reflected in the questions concerning which measures gamblers, and family members, expect the government to implement. In the Nationwide Survey, respondents are requested to reply within the context of multiple-choice answers, including: “advocate and disseminate correct knowledge and promote understanding of addiction,” “expand consulting and support services and offer locational information where people can receive help,” “increase medical agencies where gamblers and family members can receive treatment,” “support and strengthen self-help groups and private organizations,” “other,” and “nothing” (11). Such answers as the “regulation of the industry” and “limit setting of wagering money” are not included in the choices.

People tend to perceive their social world based on primary frameworks (35), where the employed framework provides a way of understanding what we experience. As Orford argues, the establishment can exercise power by choosing “which topics are discussed and which are not” (36) p. 1193. Japanese health policies have historically emphasized self-responsibility for one’s wellbeing. Recently implemented measures regarding gambling problems also reinforce this emphasis, focusing on individuals “addicted” to gambling and their family members. Within this individual pathology-oriented framework, the aim of this brief report is to show how family members of people with gambling problems relate to their situation.

The stigma associated with gambling problems has been studied previously (37), involving a social process where individuals are deeply discredited by society because of a perceived attribute or behavior (38). Stereotyping and discrimination from public stigma of the general population may lead to self-stigma, where individuals internalize and apply perceived societal conceptions to themselves (39). Compared with gamblers, family members have in research received less attention for their experiences of gambling-caused problems. To be able to design fruitful prevention and support interventions, more knowledge is needed based on accounts from family members of non-help-seeking gamblers (40). This report considers their experiences.

## Methods

This report analyzes the narratives of six family members of people with adverse gambling problems. The first author conducted two group interviews with five family members of gamblers in western Japan in October 2021. The interviews lasted 2 h each, were audio-recorded and transcribed verbatim. The group interviews included four parents and one spouse, all of whom were recruited from the members of the psycho-education seminars given by the first author. Each of the five family members is identified as A (mother, in her 70s), B (mother, in her 60s), C (father, B’s husband, in his 60s), D (wife, in her 50s), and E (mother, in her 60s). D’s husband was in treatment for gambling, but the gamblers of the other family members refused to seek help. In addition, an individual interview with participant F (mother, in her 70s) was included in the study. She did not participate in the group interviews but has been a regular participant in the psycho-educational seminars conducted by the first author. Therefore, the authors asked for her permission. She read the accounts and consented to this description by mail. All participants were informed about the aims, methods, voluntariness of participation, confidentiality and right not to answer specific questions and withdraw their consent without reprisal, how the data will be analyzed and published, possible negative consequences from participation, and contact information. All of the approached family members agreed to participate and gave their informed consent.

In the interviews, the first author strived to generate detailed accounts from the family members by using open ended questions and probes to build stories through conversations (41). The first author translated the interview transcriptions from Japanese to English. The analytical process was conducted in line with an interpretative phenomenological analysis (42), with a focus on exploring how participants make sense of their personal and social world. The transcripts were repeatedly read to familiarize with the material. Similar and convergent themes were identified within and across interviews. The emerging themes were then synthesized into three more specific themes that empirically capture what the participants wanted to share about their experience of living with someone close with gambling problems: (1) Secrecy and social isolation; (2) Expectations of providing for family members to avoid social exclusion; and (3) Self-blame and sense of failure as parents. The quotes below are examples of these specific themes apparent in the material.

## Results

### Secrecy and social isolation

The family members bear witness of secrecy to keep up appearances and avoid the public stigma of being disqualified from social acceptance in society. The situation involves psychological distress with high levels of anxiety. The burden thus not only involves financial hardships, but also consequences for mental health and family relations.

*A: I was worried about my son’s debts. I didn’t talk to anyone, not even my parents or siblings. I didn’t want anyone to ask about my son. I was scared if someone would ask about him; What should I say? My siblings never asked anything about my son; they probably knew something was wrong with my son. Sometimes, they would ask, “Is he all right?” I would then reply, “Yes, fine.” That’s all.*

*E: I feel isolated. I think my sisters probably don’t feel obliged to feel this way. Ordinary people would never understand how I felt about my son. I once wished my son were dead. Ordinary parents would not think this way.*

As revealed in Mother A’s and Mother E’s accounts above, keeping their sons’ gambling problems hidden is a heavy burden, involving lies to avoid being judged or labeled, and crucial also within the extensive family. To talk to their siblings about their children’s predicaments is not an option, which puts the women in isolated and alienated positions. Mother E even distinguish herself from “ordinary parents,” differentiating between “us” and “them,” when admitting that she once wished her son was dead.

### Expectations of providing for family members to avoid social exclusion

Despite staying abstinent for as long as a decade, gamblers can still feel ashamed for having done “stupid” things and harmed their family members. The family members, especially mothers, in turn blame themselves for having enabled their children’s gambling for a long time. Gamblers’ family members often criticize and label other family members who cannot stop bailing out their relatives, saying: “That mother is no good.”

Family members are advised not to bail out gamblers’ debts because it perpetuates the gambling. However, if debts are not paid, gamblers may lose their own families, friends or jobs. Gamblers’ family members must make the difficult choice between bailing out their family members or subjecting them to social exclusion.

*A: For five or six years, I repeatedly paid his debts, 10 million yen* (75 000 USD) *in total. I didn’t know where I could get help. I didn’t want to talk to anyone. I tried to do everything I could do within the family boundary. Keeping up appearances was most important.*

*B: About 10 years ago, my son borrowed money from my mother. Last year, another debt was discovered. The debt my mother repaid was small. This time, big money was borrowed from his cousin for other debts as well. I thought I had to pay it back as soon as possible. This cousin is very close to us.*

In everyday life, parents also encounter social pressure to bail out their children, as is revealed by Mother F. The gambler left home, angry with his mother, who refused to give him money. His mother received a notice of his tax arrears. She brought the notice to the city office, informing the staff that she was unaware of her son’s whereabouts. She asked the staff to make contact with him. The city officer asked her, “You are his mother, aren’t you?” At first, she was embarrassed, thinking that other people in the office had heard this. “I don’t want people in this office to hear this. Should I pay his taxes? That would be easier.” However, Mother F was determined not to bail out her gambling son. She repeated, “I don’t know my son’s whereabouts.” Mounted debts and uncertainty are part of the burden families carry. In addition, they risk facing frequent embarrassment in public offices.

The high expectations of taking care of family members in Japanese society is also revealed in Wife D’s case. The burden of not only taking care of her children with Autism spectrum disorders and her old mother, but also her own husband with gambling problems, becomes heavy. But Wife D believes it is expected of her and that it has been her responsibility, her “calling,” and now takes care of her gambling husband as if he were her son.

*D: I have lived my life taking care of these people (husband and children with Autism spectrum disorder). “This is my calling.” In those days, I used to believe that. (…) I now take care of my old mother, and my husband is another child—the most troublesome one.*

### Self-blame and sense of failure as parents

Non-gambling family members suffer from the results of excessive gambling by their loved ones. However, the family members do not even question the industry’s responsibility and instead blame themselves for not being good parents. Confined to the framework of individual pathology, the participants cannot relate to how gamblers’ problems are generated in the socio-political-cultural environment. Parents instead feel guilty and inadequate for failing to nurture or protect their children. When the first author asked how family members experience and perceive gambling-caused harm, Mother A responded:

*A: When I bailed out my son repeatedly, I thought I wasn’t wrong, not me, not my responsibility; my wayward son caused this mess. I have been attending meetings* (at a self-help group for family members of gamblers) *for a decade, and now I know I did something wrong.*

The first author probed further, “Have you ever thought the gambling industry was in the wrong?” “No,” the mother replied. The responsibility for the gambling problem is thus turned from her son to including also her own behavior as a mother, but placing any blame on the industry is not an alternative in her framework.

Father C believes his alcoholism led to his son’s gambling problem. He labels himself as a “worn-out and sloppy father,” which is a strong stigma to carry as a parent.

*C: I am an alcoholic, and my son inherited this predisposition. I blame myself for my alcoholism, which drove my son to gamble. This is the cycle of addiction, I believe. When my son was two years old, my wife and I divorced. When my son was in the 4th year of elementary school, my wife and I re-married. Two years after this, I relapsed. I started drinking again. When my son was a freshman in high school, I stopped drinking. During this period, my son was in a susceptible period of his life. He was exposed to a worn-out and sloppy father, me, which caused my son to gamble.*

Mother E feels guilty for failing to protect her son and remaining unaware of his gambling problems.

*E: I thought something had been wrong with my son. He didn’t pay rent or college tuition. Something was wrong, but I wasn’t aware that my son suffered from a gambling addiction. My son said, “I know what I’m doing, but I can’t stop.” I couldn’t understand him at that time. (…) The police came to us, asking about my son’s whereabouts. There was a case of theft at my son’s school. I thought I shouldn’t let my son commit another crime. I looked for my son, searching from one pachinko parlor to another, looking for my son’s bicycle. Finally, I found my son’s bicycle at an internet café. I called my husband, who told me to inform the police of my son’s whereabouts. Then the police came to the internet café. I was shocked to see my son handcuffed in front of me. On the other hand, I was relieved that my son wouldn’t commit another crime. However, even now, I’m not sure if I made the right decision.*

Mother E talks about how she still feels uncertainty and guilt after having turned her son in to the policy many years ago. The parents struggle with finding ways to relate to and handle their children’s problems. In doing so they blame themselves for being bad parents, rather than framing the problem as taking place within a broader socioeconomic context where restrictions of gambling opportunities essentially is lacking.

## Discussion

This brief report adds to the literature by showing how family members of gamblers, primarily mothers, relate to their situation within the specific individual pathology-framework as the Japanese legislation as well as public and professional perceptions adhere to. The family members experience felt stigma of being bad parents which elicits shame and efforts to maintain secrecy, as well as public stigma involving labeling, risks of status loss, social exclusion and discrimination (38). People tend to internalize the general public’s perception and act on the internalized perception. As observed among Australian gamblers (43), if the gamblers believe they are responsible for their “wrongdoings,” they will be ashamed to disclose their gambling problems. They will try to overcome gambling-caused problems without asking for outside help (27, 28). Based on the narratives of this study, shame, secrecy and stigma is not only a concern for people with gambling problems (37) but also for their affected family members (17). They are with Goffman’s words seen as “discredited person[s] facing an unaccepting world” (38) p. 31. They try to hide the problems so that they are not “disqualified from full social acceptance” (38) p. 9. To do so, they live in secrecy (17); they “pay a great psychological price, a very high level of anxiety, in living a life that can collapse at any moment” (38) p. 109, as is illustrated in the family members’ narratives.

By framing gambling problems as an individual pathology, the national gambling policies divert the attention of the Japanese public from the external gambling environment to the pathology of gamblers and their family members. The public perceives gamblers as problematic and troublesome people who have caused their predicaments on themselves (27). Thus, a pattern of blaming those who suffer from gambling is established (43). This double-bind dilemma pushes gamblers and their family members deeper into isolation.

The gamblers’ families, especially the parents, feel responsible for the gamblers’ problems; therefore, they bail out their gambling loved ones so they will not be excluded from society. Furthermore, Japanese social values and practices reinforce a sense of guilt and shame on the part of family members who feel obliged to take responsibility for the wrongdoing of another family member. As sociologist Sakuta explains: “The household has been the important institution to foster conformity to the societal demand” (44) p. 15. Japanese practices require guarantors (usually family members) when renting an apartment room or often getting a job. As the precondition for receiving welfare, the Japanese Public Assistance Act (45) requires the potential recipients to exhaust available resources, including support from persons under a duty of support (e.g., parents, siblings, children). Even if parents are not obliged to bail out gamblers, society will press them, as seen in Mother F’s experience. The negative economic effects for the family often reported in research (1, 9) might be even worse in Japan, considering that parents have to pay for their children’s college education and are expected to provide for the aging generation by taking care of their parents when they retire.

Our findings are consistent with results from a recent survey conducted in Macau (46) showing that family members of people with gambling problems are prone to emotional stress. More than 60 percent of family members reported they had helped a gambler with their problems and more than 40 percent believed they had the responsibility to pay off the debts of their family member, which points to the high social expectation of the role of parents and spouses in Asian communities. There is a great need for emotional support and debt counseling for families to turn to.

Measures to respond to gambling-related problems have been implemented recently at the municipal level in Japan, e.g., by providing group therapy for gamblers. Treatment for gamblers is included in the public health insurance since 2020 (47), but still only covers 70 percent of the total expenses. Support for family members is not included. A study conducted in Singapore suggest that family members of excessive gamblers should be educated to be able to enforce RG measures such as self-exclusion on their loved ones (48). In absence of a public health approach with general prevention measures and control of accessibility of gambling opportunities, such suggestions run the risk of adding further burden on the shoulders on parents and other affected family members.

The Japanese government claims that gambling addiction is a treatable disease, and anyone can become addicted to gambling. If gambling is so addictive, gambling products would also contribute to the addiction and thus be inherently risky. However, the research and discussion on risky gambling products and how they are provided are not included in gambling policy agendas (21, 49–51). EGM gamblers especially are encouraged to play in a *tekidoni*-like manner (moderate-like manner) without disclosing any information regarding the monetary amount that gamblers can lose for particular EGM machines per hour (52). Online casino gambling in Japan is said to be increasing rapidly partly due to the COVID-19 restrictions when people spend more time at home. Despite being illegal, access to online casinos operating from other countries is, however, ubiquitous and the legal status often unclear for the customers (53).^4^ There is thus a need for clear regulations concerning both online casinos and EGM parlors in Japan.

Treatment-seeking gamblers and their family members in Japan do not talk about prevention. The family members feel that they are “too late” for prevention. They also have no illusions of expecting the government to regulate the industry practice. The most immediate solution for treatment-seeking gamblers and their family members is to stop gambling and regain ordinary daily lives. As Orford (36) p. 1195 explains: “Grasping structural explanations for their circumstances” is difficult for them, and “the consequent tendency for [these] people [is] to blame themselves and to feel shame and responsibility for the harm they experience.” More attention in policy and research needs to be directed toward addressing the social and cultural context in which gambling takes place, i.e., the availability of gambling (18, 54), as well as on the extensive harms for families and communities (1, 55–58).

## Conclusion

Numerous gambling-caused problems are framed as the problem of gamblers and their family members, consequently provoking the public to focus on the pathology of gamblers and their family members. Socio-political-cultural factors are excluded from the public’s perspective. Gamblers and their families are ashamed to disclose their problems and, thus, do not seek help. Recently, growing criticism has emerged for this narrow perspective (2, 49, 51) that places the burden of gambling disorders on individual gamblers and their families. It is necessary to shift the focus of gambling policies from individual pathologies to socio-political-cultural factors, investigating how these factors influence gambling-caused harm, especially in the Japanese context.

In this report, the focus on individual pathology in Japanese legislation and public and professional perception has been illustrated and contrasted with the lived experiences of gamblers’ family members. It brings insights into the inconsistencies and unreasonableness of the “responsible gambling” paradigm originating from the Reno model. However, the report is preliminary in nature since interviewing more family members could have offered more robust findings. Furthermore, in future research, the discourses adopted in psychological aspects of individuals and inter-personal relations such as “distorted thinking” and “co-dependency” must be examined as to how the treatment methods and practices influence the perception of gambling-caused problems on the part of gamblers, family members, treatment, welfare service providers and the general public.

## Data availability statement

The datasets presented in this article are not readily available because due to the sensitive nature of this research, participants of this study did not agree for their data to be shared publicly.

## Ethics statement

This study was reviewed and approved by the Otani University Research Ethics Committee. The participants provided their written informed consent to participate in this study.

## Author contributions

NT conducted interviews with family members. All authors contributed equally to the analysis and writing of this report.

## Footnotes

1 The forms of gambling spent most money on among gamblers who scored SOGS5+: pachinko 38.7%, pachislot 32.3%, horse betting 11.0%, and lotteries 7.1% according to most recent national prevalence survey in 2021 (11).

2 Since the legal status of *pachinko* and *pachislot* is that of gambling, *pachinko* and *pachislot* addiction is legally excluded from gambling addiction. Therefore, the phrase “so forth” is used to refer to *pachinko* and *pachislot* addiction.

3 The English version of the Health Promotion Act is adopted from the Japanese Law Translation (32).

4 Recently a man was arrested in Japan for fraud after mistakenly have been transferred 46.3 million yen (340 000 USD), money intended for households’ benefits. The man said to have spent the entire amount at overseas online casino (53).
